# Effects of mammalian herbivore declines on plant communities: observations and experiments in an African savanna

**DOI:** 10.1111/1365-2745.12096

**Published:** 2013-06-06

**Authors:** Hillary S Young, Douglas J McCauley, Kristofer M Helgen, Jacob R Goheen, Erik Otárola-Castillo, Todd M Palmer, Robert M Pringle, Truman P Young, Rodolfo Dirzo

**Affiliations:** 1 Division of Mammals, Smithsonian InstitutionWashington, DC, 20013, USA; 2 Department of Biology, Stanford UniversityStanford, CA, 94305, USA; 3 Mpala Research CentreBox 555, Nanyuki, Kenya; 4 Harvard University Center for the EnvironmentCambridge, MA, 02138, USA; 5 Department of Environmental Science, Policy, and Management, University of California at BerkeleyBerkeley, CA, 94720, USA; 6 Department of Zoology and Physiology, University of WyomingLaramie, WY, 82071, USA; 7 Department of Human Evolutionary Biology, Harvard UniversityCambridge, MA, 02138, USA; 8 Department of Ecology, Evolution and Organismal Biology, Iowa State UniversityAmes, IO, 50010, USA; 9 Department of Zoology, University of FloridaGainesville, FL, 32611, USA; 10 Department of Ecology and Evolutionary Biology, Princeton UniversityPrinceton, NJ, 08544, USA; 11 Department of Plant Sciences, University of CaliforniaDavis, CA, 95616, USA

**Keywords:** abiotic gradients, community structure, East Africa, exclosure experiment, herbivory, livestock–wildlife interactions, plant species richness, plant–herbivore interactions, wildlife decline

## Abstract

**1.** Herbivores influence the structure and composition of terrestrial plant communities. However, responses of plant communities to herbivory are variable and depend on environmental conditions, herbivore identity and herbivore abundance. As anthropogenic impacts continue to drive large declines in wild herbivores, understanding the context dependence of herbivore impacts on plant communities becomes increasingly important.

**2.** Exclosure experiments are frequently used to assess how ecosystems reorganize in the face of large wild herbivore defaunation. Yet in many landscapes, declines in large wildlife are often accompanied by other anthropogenic activities, especially land conversion to livestock production. In such cases, exclosure experiments may not reflect typical outcomes of human-driven extirpations of wild herbivores.

**3.** Here, we examine how plant community responses to changes in the identity and abundance of large herbivores interact with abiotic factors (rainfall and soil properties). We also explore how effects of wild herbivores on plant communities differ between large-scale herbivore exclosures and landscape sites where anthropogenic activity has caused wildlife declines, often accompanied by livestock increases.

**4.** Abiotic context modulated the responses of plant communities to herbivore declines with stronger effect sizes in lower-productivity environments. Also, shifts in plant community structure, composition and species richness following wildlife declines differed considerably between exclosure experiments and landscape sites in which wild herbivores had declined and were often replaced by livestock. Plant communities in low wildlife landscape sites were distinct in both composition and physical structure from both exclosure and control sites in experiments. The power of environmental (soil and rainfall) gradients in influencing plant response to herbivores was also greatly dampened or absent in the landscape sites. One likely explanation for these observed differences is the compensatory effect of livestock associated with the depression or extirpation of wildlife.

**5.** *Synthesis*. Our results emphasize the importance of abiotic environmental heterogeneity in modulating the effects of mammalian herbivory on plant communities and the importance of such covariation in understanding effects of wild herbivore declines. They also suggest caution when extrapolating results from exclosure experiments to predict the consequences of defaunation as it proceeds in the Anthropocene.

## Introduction

Populations of wild large herbivores are declining throughout much of the world (Collen *et al*. 2009; Wilkie *et al*. 2011). Such herbivores have strong effects on the composition, richness, physical structure and successional patterns of plant communities across multiple biomes and continents (Knapp *et al*. 1999; Bakker *et al*. 2006; Beguin, Pothier & Cote 2011), and changes in their abundances can lead to dramatic direct and indirect effects on plant–animal interactions and ecosystem processes. However, the magnitude and direction of the effects of herbivores on plant communities are variable (Vesk & Westoby 2001). With respect to plant diversity or species richness, the loss of mammalian herbivores has been shown to have positive (Collins *et al*. 1998), negative (Dirzo & Miranda 1992; Proulx & Mazumder 1998), neutral (Adler *et al*. 2005) and mixed effects (Kohyani *et al*. 2008) in different systems. Variation in abiotic gradients (McNaughton 1983; Gough & Grace 1998; Augustine & McNaughton 2006; Bakker *et al*. 2006; Hillebrand *et al*. 2007) and the degree of compensation by livestock (or other) herbivores (Young, Palmer & Gadd 2005; Veblen & Young 2010) probably mediate the plant community response to the loss of wild herbivores, although simultaneous examination of these factors (and their interactions) has received scant attention.

Several studies have suggested that herbivores should increase plant diversity in high-productivity conditions and decrease it at low productivity (Olff & Ritchie 1998; Proulx & Mazumder 1998; Bakker *et al*. 2006). However, the environmental attributes that mediate these interactions are variable across sites, and independent variation in environmental gradients can lead to divergent effects on the magnitude of plant community response to herbivore declines or exclusions (Augustine & McNaughton 2006; Anderson, Ritchie & McNaughton 2007).

Livestock may have overall effects on plant diversity that are similar to those of wild large herbivores (Olff & Ritchie 1998). Yet, there are also substantial differences between the diets, behaviour and sometimes densities of livestock and those of large wildlife, which can influence their respective effects on plant communities and successional patterns (Vázquez & Simberloff 2004; Riginos & Young 2007; Riginos *et al*. 2012). For example, the replacement of wild browsers by livestock may alter competition and facilitation among plant communities (Veblen & Young 2010), patterns of tree recruitment (Tobler, Cochard & Edwards 2003; Goheen *et al*. 2010) and nutrient distribution (Augustine 2003). In particular, megaherbivores, such as elephants, have strong direct impacts on woody vegetation via browsing or physical damage, with subsequent indirect impacts on plant and faunal communities (Pringle 2008); livestock are unlikely to compensate for these roles. Such differences in function and size of herbivores are known to mediate herbivore effects in other systems (Bakker *et al*. 2006), and it thus seems likely that there will be complex interactions between the replacement of large wild herbivores with livestock and the underlying abiotic gradients on characteristics of plant communities.

Much of the experimental work on the effects of wildlife extirpations on plant communities has been conducted using exclosure experiments. Ecologists often employ exclosure experiments as surrogates for areas from which wild herbivores have been extirpated and compare such manipulations to control sites at which wild herbivores are still abundant (Terborgh & Wright 1994; Asner *et al*. 2009; Ripple, Rooney & Beschta 2010). While such experiments are vital to isolating the impacts of wildlife on ecological processes, the extent to which they can be generalized to characterize the complex changes that often accompany wildlife declines following anthropogenic disturbance is unclear. Other studies have shown that experimental ecological manipulations often do not accurately mimic those occurring via anthropogenic disturbances (Skelly 2002). Through exclosure experiments, wildlife presence typically is manipulated in isolation of other factors, whereas wildlife loss in other contexts is often accompanied by a suite of additional changes in human activities. For example, the decline of large mammals in rangelands is often associated with (and hastened by) their replacement by livestock such as cattle, sheep and goats (Loft, Menke & Kie 1991; Prins 1992; Du Toit & Cumming 1999). Therefore, it is important to understand when (and ideally why) experimental exclusions do or do not mimic ecological changes that occur across landscapes where large mammals are in decline due to the multifaceted activities of humans.

In this study, we examined the interactive roles of soil, rainfall and livestock in modulating the response of local plant communities to large wildlife declines. We investigated these interactions within a small geographic area, set across steep rainfall and soil texture gradients, drawing on both exclusion experiments and more typically disturbed areas throughout the region, where large wildlife declines were often associated with increases in livestock abundance. Unless otherwise stated, hereafter the terms ‘wildlife’ and ‘wild herbivores’ are used to denote large (> 5 kg), herbivorous wild mammals.

This research was conducted in the savannas of Laikipia County of Kenya, East Africa, where we tested the following hypotheses: (i) soil characteristics and rainfall influence the strength and direction of effects of wild herbivores on plant species richness, species and growth form composition, and physical structure (height, cover, aerial cover). (ii) Effects of declines of large wild herbivores on plant communities are stronger in exclosure experiments (‘experimental sites’ hereafter) where wildlife declines occur in isolation than in the broader landscape, where defaunation is often accompanied by replacement with livestock (‘landscape sites’ hereafter). (iii) There are interactions between the effect of experimental vs. landscape context and the role of environmental gradients in determining effects of large herbivore declines on plant communities, with stronger effects of environmental gradients predicted in experimental sites. (iv) Because compensation by livestock for wildlife declines is probably not complete, variation in effects of wildlife decline between experimental and landscape sites is not fully accounted for by total stocking density of herbivores.

## Materials and methods

### Study system

Our research was conducted across 74 sites in semi-arid savanna woodland in Laikipia District (1700–1800 m a.s.l., 36°52′ E, 0°17′ N) between January and July 2011. This study area encompasses (i) strong gradients in rainfall and soil attributes; (ii) varying levels of large wildlife and livestock abundances throughout these abiotic gradients and (iii) several wildlife exclosure experiments that span both of these abiotic gradients. We are thus able to compare results of experimental large wildlife removals to those of anthropogenic large wildlife declines that are associated with secondary replacement by livestock.

Common land uses in Laikipia include wildlife-only conservancies, pastoral rangelands, mixed conservancy and livestock production (of differing intensities) and agriculture. Wildlife densities are greatest in conservancies and generally decrease with intensifying livestock production. Croplands were excluded from this study. Wildlife in this region includes a variety of both migratory and sedentary species across a broad range of size classes (Kinnaird & O'Brien 2012).

**Fig. 1:**
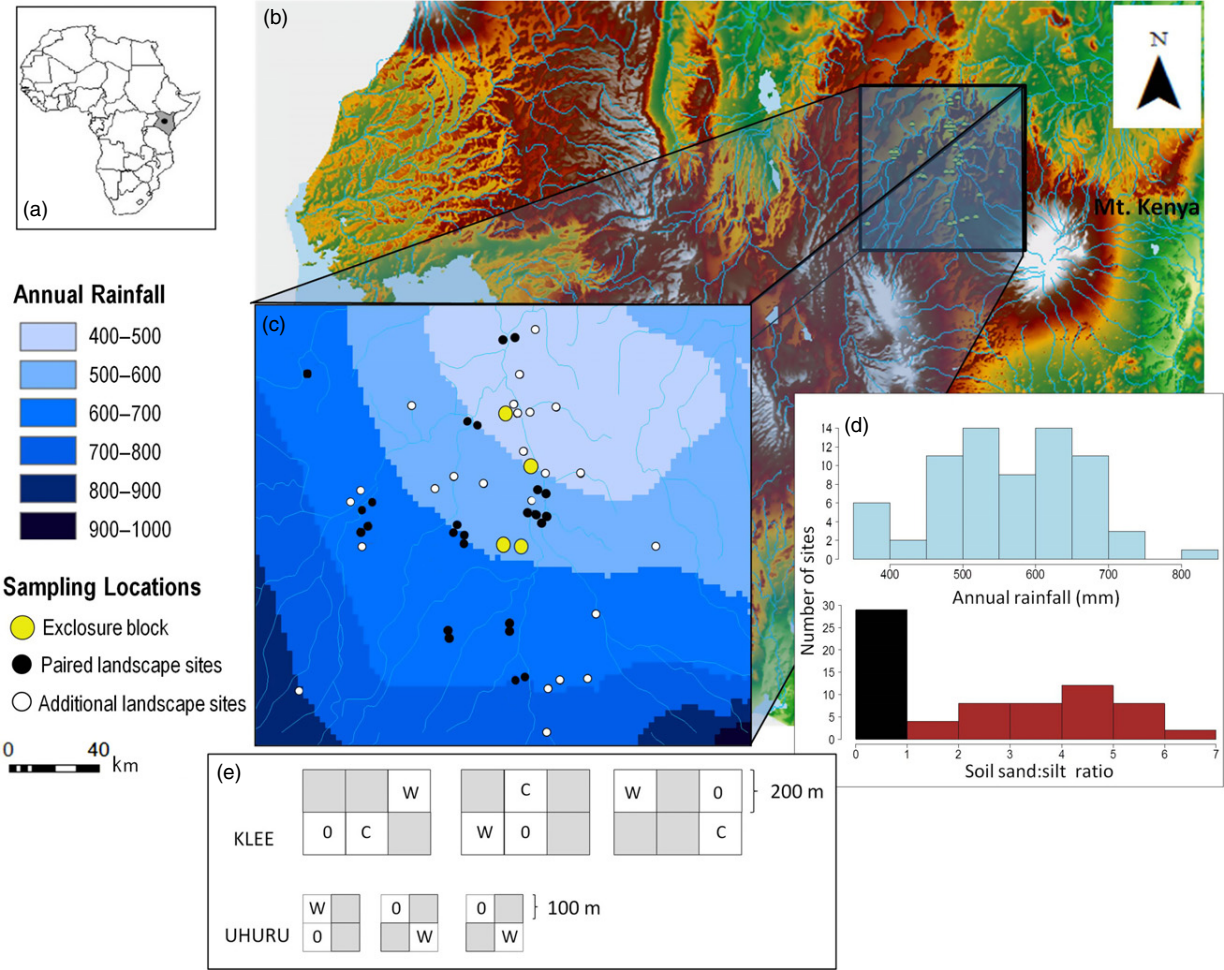
The study area, located in the Laikipia County of central Kenya (a), shown in topographic detail (b). The 74 sampling sites are located across a strong rainfall gradient. The sampling sites included 27 ‘experimental’ sites, arranged in blocks and located across two different large-scale exclosure experiments (yellow circles), and 47 ‘landscape’ sites (see text for details). Landscape sites that were analysed using a paired design are shown in black, and additional landscape sites are in white. Sites are distributed normally across the rainfall gradient (c) and bimodally across an underlying soil gradient (d) including predominantly high-clay black cotton soils (black bars), and a continuum of transitional and red sand soils (red bars). Panel e shows the randomized block design (1 triplet block per experiment shown) used in the two experiments (KLEE and UHURU). Each experiment includes treatments that allow access to all animals (0), wildlife (W) or cattle (c; KLEE only) as well as other treatments (not used; shown in grey); distance between replicate blocks is not to scale.

Two broadly defined soil types underlie most of Laikipia and are widespread throughout East Africa. Black cotton soils (pellic vertisols) are of recent volcanic origin, are highly productive, have high concentrations of clay and silt and are characterized by poor drainage and pronounced shrink–swell dynamics. Red sand soils (ferric and chromic luvisols) are sandy, friable loams of metamorphic origin and typically support plant communities lower in primary productivity than black cotton soils (Augustine & McNaughton 2006; Pringle *et al*. 2007). Intermediate and transitional soil types (including aspects characteristic of both red and black cotton soil types) also exist. Therefore, instead of using a categorical definition of soils, we assessed soil type as a continuous metric of the ratio of sand to silt in soils. We found a *c*. 9-fold variation in sand:silt ratio across sites. The distribution of sites across this soil gradient was bimodal, coinciding with the two broad soil types characteristic of the area (see Fig. 1C). Of the 74 sites, 27 were part of manipulative large-herbivore exclosure experiments (details below), while the remaining 47 study sites were spread throughout the district (details below), so as to encompass sites with a broad range of wildlife and livestock abundances and abiotic variables. All sites were 1 ha in size (100 × 100 m).

### Experimental sites

To examine experimentally the effects of wildlife declines on plant communities, we surveyed vegetation in two different experimental sites: the Kenya Long-term Exclosure Experiment (KLEE, established in 1995; Young *et al*. 1998) and the Ungulate Herbivory Under Rainfall Uncertainty experiment (UHURU, established in 2008; Goheen *et al*. 2013). Both experiments employ a similar block design in which each block includes three total exclosure sites from which all large wildlife is excluded using electric fences (for UHURU, this comprises all animals > *c*. 5 kg, while for KLEE, this effectively excludes only animals larger than > 15 kg) paired with three open sites, which allow access to large herbivores. Sites in KLEE are 4 ha in size, of which we sampled only the central 1 ha, while sites in UHURU are each 1 ha. In both of these experiments, other manipulative treatments selectively permit access to different size classes of wild mammals. In KLEE (but not in UHURU), an additional cattle-only treatment exists, which permits cattle only at low to moderate stocking intensities (Young *et al*. 1998; Young, Palmer & Gadd 2005). Our sampling was conducted only in total exclosures (no wildlife or cattle), control sites (all herbivores allowed) and cattle-only sites within KLEE. Paired analyses compared full exclosure to control plots (cattle plots not included). KLEE is located on black cotton soils, and UHURU is located on red sand soils.

### Landscape sites

Our 47 landscape sites were spread over a *c*. 3000 km^2^ of Laikipia and were selected to encompass (i) sites across the rainfall and soil texture spectrum and (ii) sites from across the range of the aforementioned land-use types, but ranging from conservancies without livestock to intense pastoral use with multiple species of livestock. These landscape sites were interspersed around the area in which the experimental sites were located (Fig. 1). Whenever a landscape site was selected in close proximity to a site with divergent management practices (i.e. a conservancy site abutted a high intensity pastoral group ranch), and there was a clear boundary separating the two land uses (i.e. river or livestock fence), we also surveyed the adjacent land parcel (both sites were located at least 100 m from the boundary; Fig. 1). For these paired landscape sites (*n* = 12 landscape site pairs; a subset of the total landscape 47 sites), we were able to conduct secondary pair-wise comparisons of relationships between wildlife status and soil nutrients, while controlling for variation in rainfall and soil type. For these paired sites, we confirmed our *a priori* designations of landscape sites as being ‘high wildlife’ or ‘low wildlife’ using dung surveys (defined in ‘Wildlife and livestock abundance’ below). Each landscape site or site pair was > 2 km from the nearest other site or site pair.

### Rainfall

Both experimental and landscape sites were selected to encompass gradients in both rainfall (from *c*. 300 to *c*. 900 mm year^−1^) and soil (low to high sand:silt ratio). Mean annual rainfall was interpolated from data gathered from rain gauges at 75 locations across the district over periods of up to 50 years (details in Franz *et al*. 2010). Rainfall in the region is highly variable; to control for effects of variation in recent rainfall on vegetation communities, we included, in all analyses, a variable of ‘recent rainfall’ in addition to the annual rainfall metric. Recent rainfall was assessed as mean rainfall across all sites where month-by-month data were available for the 3 months prior to the survey date. This metric was included to account for seasonal variation in rainfall across the sampling period, which could affect plant responses, particularly structural metrics. Average annual rainfall was distributed normally across the sites (Fig. 1D). The three exclosure/control/cattle-only triplets in KLEE all receive similar rainfall (< 5% variability among sites), while the 9 UHURU site pairs span a strong rainfall gradient (three pairs set at each of three locations distributed along the rainfall gradient).

### Vegetation surveys

At each experimental and landscape site, we established a grid of 50 sampling points (with 20 × 20 m spacing between grid points). At each point, we dropped five sample pins (50 cm in height), each 1 m apart (total 250 pins per grid). For each pin drop, we recorded the identity of all plant species touching the pin, as well as the height at which the individual touched the pin. We also recorded the presence of all plant species directly above the pin that exceeded 50 cm in height. Because some plants could not be identified to species, particularly when not in flower, these species were aggregated at the genus level to minimize effects of recent rainfall on species richness assessments per site. This issue arose in < 10% of all measurements. Additionally, for both structural (mean vegetation height, per cent aerial cover and total cover) and species richness estimates, we conducted a secondary analysis only on paired sites (high wildlife and low wildlife, sampled simultaneously), as a second approach to minimize the possible influence of spatio-temporal variation on effects observed.

Species composition data were analysed based on aerial cover of each species (the per cent of 250 pin drops per site, which a given plant species touched) within a site. All physical structure and species richness data were pooled at the site level, and analyses were conducted with site (or site pair in paired analyses) as the unit of analysis. We used maximum height of herbaceous vegetation (highest point of contact of vegetation with the pin), total cover (the average number of contacts of vegetation per survey pin, which can exceed 100%) and per cent aerial cover (per cent of pins touching any vegetation) as metrics of physical structure.

### Wildlife and livestock abundance

At each sampling point (50 per site), we surveyed the abundance (% cover) of dung within a 1 m^2^ area surrounding the point. We identified each dung pile to the lowest taxonomic level possible (usually species); however, for the purposes of these analyses, species were pooled as ‘domestic’ or ‘wildlife’ dung. To validate the utility of this metric in estimating wildlife and domestic stock abundance, we compared dung surveys to camera-trap surveys conducted simultaneously at each of the sites, finding consistent results (Appendix S1, Fig. S1 in Supporting Information).

For comparison of paired sites (see Landscape sites above), we verified differences in wildlife abundance across site pairs using dung surveys. All high wildlife sites had higher than median levels of wildlife dung cover across all sites, while all low wildlife sites were defined as having wildlife dung cover in the bottom quartile of wildlife dung cover. Sites with intermediate levels of dung were not part of any paired analyses. To account for potential effects of wildlife on soil properties (e.g. McNaughton, Banyikwa & McNaughton 1997), we also examined the differences in soil properties among paired sites (Appendix S2).

### Statistical analyses

We examined effects of variation in wildlife abundance on plant community structure and species richness using Generalized Linear Models (GLMs). GLMs were constructed using Poisson errors and log-link functions for analysis of species richness and using Gaussian errors and identity link functions for analyses of per cent aerial cover, total cover and mean vegetation height. For each modelled response, we first constructed sets of candidate regression models in statistical software r v 2.14.2 (R Development Core Team 2012) using the following variables as factors: wildlife abundance (estimated by per cent wildlife dung cover), livestock abundance (estimated by per cent livestock dung cover), mean annual rainfall, recent rainfall in 3 months prior to the survey, soil sand:silt ratio and a categorical classification of ‘experimental status’ as ‘experimental’ for experimental sites (both exclosures and their paired controls) and ‘landscape’ for all other sites. Candidate models included all interactions between wildlife abundance and livestock abundance with soil, annual rainfall, and experimental status and the interaction of wildlife and livestock abundance. To compare effects of changes in wildlife abundance as opposed to all herbivore abundance, similar models were also run (separately) with all herbivores (and all interactions between herbivore abundance and abiotic gradients) rather than with wild herbivores and livestock separated. Tables with these (all herbivores) results are reported as supplementary tables and not in main text except where specified.

To focus directly on the effects of wildlife decline and interactions with abiotic gradients, we performed a second analysis of effect size on the response of vegetation to wildlife declines using only the subset of sites that were (i) spatially paired across different land-use types and (ii) sampled simultaneously (*n* = 24 pairs, 12 landscape pairs and 12 experimental pairs). Effect sizes between high wildlife and low wildlife designations (i.e. our two categories of landscape sites) were calculated using the formula ln (*plant metric*_low wildlife_/*plant metric*_high wildlife_) (Hedges, Gurevitch & Curtis 1999). We examined drivers of this effect size using GLMs (as described above) with the factors experimental status, annual rainfall, soil sand:silt ratio and the interactions of these two abiotic variables with experimental status. Based both on data structure and on biological evidence for nonlinear relationships between productivity and plant responses to herbivore removal, we applied models that included a square-transformed rainfall term as well as models that included only a linear rainfall term. A square-transformed soil term (sand:silt) was initially included but was dropped due to lack of support in the models. Prior to all GLM analyses, we tested all factors and found no substantial colinearity (VIF < 2) using variance inflation factors.

We compared all possible GLM models using AIC_c_ and Akaike weights (Burnham & Anderson 2002). Because there were multiple candidate models that received substantial empirical support (AIC_c_ < 2), we used model averaging to more directly compare competing models. With this approach, we calculated model-averaged parameter estimates for all models with ΔAIC_c_ < 2, with each model's contribution to parameter estimates being proportional to its Akaike weight (MuMIN, Barton 2009). To visualize the responses of plant community data, linear regressions and partial residual plots were created based on best-fit parameters across all the averaged model parameters.

To examine changes in plant community composition and growth form (all species classified as either forb, grass, sedge, succulent and woody) as a function of wildlife abundance, livestock abundance and abiotic factors, we used nonmetric multidimensional scaling (NMDS) on both the species-level composition data and on life-form composition. We analysed the effects of wildlife abundance and environmental factors on plant community composition NMDS results using nonparametric multivariate anovas (npmanova; McArdle & Anderson 2001) and calculated *P* values using general permutation procedures (Manley 2006). We also compared best-fit models of plant community species and growth form responses using a multivariate AIC. We used the following reduced set of main factors: wildlife abundance, annual rainfall, soil sand:silt ratio and a categorical classification of experimental vs. landscape treatments. We considered all interactions of wildlife abundance with soil, annual rainfall and experimental status. As the best-fit model for both growth form and species composition had much stronger support than competing models (ΔAIC_c_ > 4), we present only the best model (r package vegan, v. 2.0–3, Oksanen *et al*. 2012). A summary of analytical approaches is provided in Table S1.

## Results

### Plant community structure

**Fig. 2:**
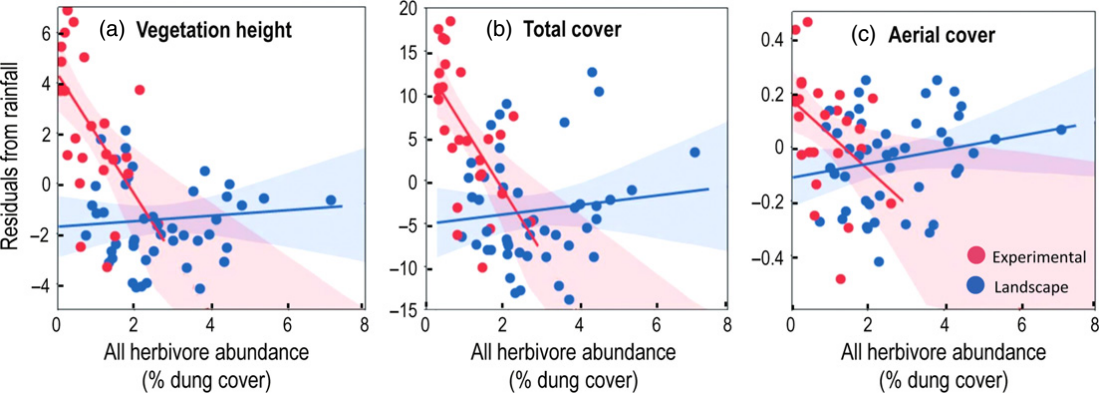
Residual variation from the regression of mean vegetation height (a), total cover (b) and aerial cover (c) to rainfall, plotted against% dung cover (a proxy for total herbivore abundance). In experimental sites (red), the removal of herbivores leads to plant communities that are taller, with more cover, and more structural complexity, after correcting for rainfall effects (all relationships significant). In contrast, in landscape sites (blue), the loss of herbivores has little effect on vegetation structure (no significant relationships).

All three plant structural metrics (mean vegetation height, per cent aerial cover and total cover) were explained by similar explanatory factors, namely wildlife abundance, annual rainfall, experimental status (experimental versus landscape site) and the interaction between wildlife and experimental status (Table 1). Rainfall, in particular, had a strong influence on vegetation structure, driving consistent increases in all structural metrics. The effect of wildlife on vegetation structure was more complex, and the effects were different in experimental and landscape sites. These effects are essentially the same whether we consider the relationship between plant community metrics and all herbivores (Table S2), or consider just wild herbivores (Table 1; Fig. S2). Total cover, aerial cover and mean vegetation height all increased strongly with decreasing abundance of herbivores in experimental sites, but had no relationship with decreasing herbivore abundance in the surrounding landscape sites (Fig. 2; all herbivores shown). In analyses that isolated the effects of wildlife and livestock, both wildlife and livestock were important in explaining plant responses. Livestock in particular was negatively correlated with both vegetation height and total cover, but minimally so with aerial cover. For all three responses, the best-fit models were able to explain a large proportion of the variance: 63% of variance was explained for total cover, 50% for aerial cover and 47% for average height (Table 1).

**Table 1:** Model average parameter estimates including standard errors (SE), relative variable importance (Rel imp) and estimated *P* values

	Vegetation Height			Aerial Cover			Total Cover		
			
	Coefficient ± SE	Rel imp	Pr(>z)	Coefficient ± SE	Rel imp	Pr(>z)	Coefficient ± SE	Rel imp	Pr(>z)
Main effects									
Wildlife	−3.448 ± 1.083	1.00	**< 0.01**	−0.1378 ± 0.0776	1.00	0.08	−7.013 ± 2.053	1.00	**< 0.001**
Experimental	−5.698 ± 1.382	1.00	**< 0.001**	−0.3010 ± 0.0918	1.00	**< 0.01**	−13.69 ± 2.956	1.00	**< 0.001**
Annual rainfall	0.016 ± 0.003	1.00	**< 0.001**	0.0014 ± 0.0003	1.00	**< 0.001**	0.039 ± 0.009	1.00	**< 0.001**
Livestock	−1.014 ± 0.578	0.95	0.08	−0.0439 ± 0.0315	0.54	ns	−2.21 ± 1.141	0.95	0.06
Soil (sand:silt ratio)	−0.160 ± 0.323	0.15	ns	−0.0067 ± 0.0246	0.24	ns	−0.413 ± 0.678	0.19	ns
Recent rainfall	−0.002 ± 0.005	0.16	ns	−0.0004 ± 0.0003	0.44	ns	−0.009 ± 0.011	0.22	ns
Selected interactions									
Experiment × wildlife	3.655 ± 0.951	1	**< 0.001**	0.1932 ± 0.058	1.00	**< 0.001**	7.648 ± 1.988	1.00	**< 0.001**
Livestock × wildlife	−0.968 ± 0.779	0.37	ns	−0.0198 ± 0.0502	0.09	ns	−1.506 ± 1.653	0.23	ns
Rainfall × wildlife	0.002 ± 0.003	0.16	ns	−0.0002 ± 0.0002	0.24	ns	0.001 ± 0.005	0.07	ns
Soil × wildlife	–	–	–	0.0103 ± 0.0072	0.11	ns	–	–	–

Analyses are based on the entire data set of 74 sites, with plant data pooled at the site level. *P* values are determined using backwards stepwise regression from the full model. No models that included soil x wildlife interactions received substantial support (AICc < 2) for height and total cover responses; thus, no values are provided. Values with significance < 0.05 are indicated in bold.

Comparisons of the subset of the 48 paired sites also revealed effects of environmental parameters to herbivore decline on plant structural response in all three structural responses (Table S2 and Fig. S3). Plant structural responses to wildlife declines were generally stronger in soils with high sand:silt ratios (red soils, Fig S3). However, for total cover and aerial cover, there was an important interaction between experimental status (experimental versus landscape sites) and soil properties, with only experimental sites showing the pattern of stronger responses in high sand:silt sites (Fig. S2; landscape sites showed no significant variation in response along the soil gradient). Responses to rainfall were u-shaped in landscape sites (lower response at intermediate levels of rainfall) and were slightly lower with increasing rainfall in experimental comparisons. The interaction between rainfall and experimental status was stronger than that between soil and experimental status for aerial cover and vegetation height, but the inverse was true for total cover.

### Species richness

In total, we recorded 137 species of plants in 48 families; of these species, 15% were found only in high wildlife sites and 9% were found only in low wildlife sites. Richness was highly variable across experimental and landscape sites, ranging from 8 to 44 species surveyed per site (SD = 8.2). GLMs of species richness across all sites explain a relatively small proportion of total variance (35% explained in best model). In contrast to structural metrics, rainfall had little effect on richness or on the magnitude of plant richness response to wild herbivore declines. Sand:silt ratios were important in determining richness; sites with high sand:silt ratios generally had overall higher richness levels (Fig. S3A). There was no effect of wildlife abundance alone on species richness across all sites (even when accounting for soil properties); however, increased abundance of all herbivores, or of livestock alone, drove declines in species richness, once variation due to soil parameters was accounted for (Fig. S3B).

**Fig. 3:**
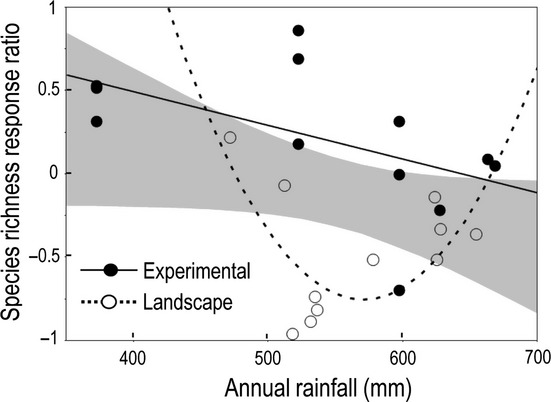
Effect sizes (log_e_ response ratios) of wildlife removal on plant species richness across a gradient of rainfall (mean annual rainfall). The effects of wildlife loss on species richness are u-shaped in landscape sites (dashed line, unfilled circles; *P* = 0.02, *R*^2^ = 0.33), and linear in experimental sites (solid black line, filled circles; *P* = 0.07, *R*^2^ = 0.21). The grey shaded area represents the 95% confidence interval across all site pairs. Only the subset of sites that were spatially and temporally paired (*n* = 24) were used for these analyses. Model details in Table S2.

Models for effect size of wildlife on only the subset of paired sites explained a greater proportion (60%) of the total variance in species richness. In these models, experimental status was the best predictor of effect size with wildlife declines leading to strong declines in plant species richness in experimental but not landscape sites (Table S3). There was also substantial support for wildlife decline driving stronger reductions in plant species richness in low rainfall environments, and some support for the effects of soil (stronger effects in high sand:silt environments) on species richness responses. Finally, there were important interactions between experimental status and rainfall on plant species richness, with generally a stronger and more linear role for environmental factors in mediating species richness responses in experimental as compared to landscape sites (Fig. 3). Soil interactions with experimental status, while present, were much more limited (Table S3).

### Composition

**Fig. 4:**
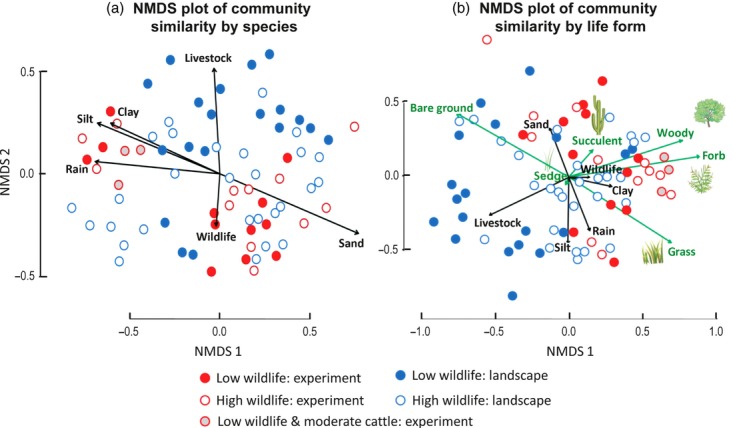
Results from discrimination analysis of plant community species composition (a) and growth form composition (b) show strong and consistent differences in composition between high wildlife (unfilled circles) and low wildlife sites (filled circles) in landscape sites (in blue) but not in experimental sites (in red). Important environmental drivers of plant community composition (shown with black arrows) are underlying soil parameters (% sand, silt and clay), annual rainfall and domestic livestock. The length of the arrow is proportional to the strength of the correlation. In panel b, the underlying patterns of growth form variation driving the differences are plotted in green arrows and green text.

The relationship between environmental variables and plant species composition and growth form composition also varied between experimental and landscape sites. Discrimination analysis found no relationship between variation in plant species community composition from high wildlife to low wildlife sites within experiments (*F* = 0.88, *R*^2^ = 0.04, *P* = 0.63), but a significant (although small) effect of this change in wildlife abundance in landscape sites (F = 2.51, *R*^2^ = 0.06, *P* < 0.0001; Fig. 4a). The NMDS analyses of composition accounted for more than 95% of variation in the first two axes (final stress = 18.44). There were strong relationships between annual rainfall (*R*^2^ = 0.55, *P* < 0.0001), soil properties (clay *R*^2^ = 0.39, *P* < 0.001; sand *R*^2^ = 0.37, *P* < 0.0001) and abundance of livestock (*R*^2^ = 0.32, *P* < 0.0001) on plant community composition as described by the first two axes of the NMDS. Consistent with findings related to species richness, wildlife itself played a relatively small role in explaining variation in plant community composition (*R*^2^ = 0.07, *P* = 0.09) in landscape sites. The best-fit models by AIC were consistent with these results, including all main effects, but also indicated the importance of an interaction of annual rainfall and wildlife abundance.

Results were similar for growth form analyses. There were significant differences between high wildlife and low wildlife sites in landscape sites, but not in exclosure experiments (Fig. 4b). The NMDS accounted for 96% of variation in growth form on the first two axes (final stress = 22.05). There were strong effects of silt (*R*^2^ = 0.15, *P* < 0.01) and sand (*R*^2^ = 0.13, *P* = 0.04) but not clay (*R*^2^ = 0.02, *P* = 0.51) on growth form composition, with grasses in particular being more common on high silt and low sand sites. Wildlife alone played a relatively small role in driving the model (*R*^2^ = 0.04, *P* = 0.22). Instead, livestock was the most significant correlate of growth form composition change (*R*^2^ = 0.29, *P* < 0.0001), with high livestock sites having particularly low levels of forbs and woody plants. Annual rainfall was also correlated with growth form composition change, with more grass and fewer succulents in high rainfall sites (*R*^2^ = 0.10, *P* = 0.02). The best-fit model determined by AIC again provided similar results and included all main effects (soil sand:silt ratio, wildlife, experimental status and annual rainfall); here the best-fit model also included the interaction between wildlife and soil. In both life-form and species-level analyses, cattle-only exclosure plots clustered with other experimental plots and differed from responses seen in landscape low wildlife sites (Fig. 4a).

## Discussion

### Effects of herbivores and environmental gradients on plant communities

As expected, both the abundance of herbivores and the underlying environmental gradients had strong impacts on plant community structure, composition and species richness. High wildlife sites had reduced vegetation cover (both aerial and total cover), shorter vegetation, more diverse communities and distinct plant species assemblages as compared to low wildlife sites. High rainfall systems and systems with low sand:silt ratios (typical of more productive black cotton soils) had higher aerial cover, more total cover and higher mean vegetation height. Plant community composition and growth form composition also diverged strongly on these gradients, with stronger dominance of grasses and reduced presence of succulents in more productive soils, and higher rainfall environments (Fig. 3).

We hypothesized that these environmental gradients should also drive variation in magnitude and direction of plant community response to herbivores (Milchunas & Lauenroth 1993; Olff & Ritchie 1998). Herbivores are thought to change diversity in different ways depending on site productivity. In high-resource environments, where light is often limiting, herbivores may increase diversity by selectively removing competitive dominants and changing microsite conditions to allow germination and the establishment of more species (Huston 1994). However, in many low-resource environments, competition generally occurs below-ground, and large herbivores may have less direct impacts (Osem, Perevolotsky & Kigel 2002). Thus, we expected to see stronger positive effects of herbivores on diversity when environmental stress was low (i.e. high rainfall, high-productivity environments) (Bakker *et al*. 2006). However, we instead saw dampened effects of herbivory on various plant metrics, including species richness, in high rainfall, high-productivity (low sand:silt) environments (Table 1), consistent with other work in this system, which has shown stronger cascading effects of herbivores on consumers in low rainfall environments (Pringle *et al*. 2007). Unique properties of higher productivity black cotton soils may also contribute to this response, since they are characterized by stressful shrink–swell dynamics. In addition, less palatable species may be more abundant on black cotton soils, dampening the response in these systems (see e.g. Goheen & Palmer 2010).

### Experimental status

Results from exclosure experiments are likely to extrapolate best to plant community responses in environments where wildlife declines are the primary form of disturbance, for example, in protected areas (Craigie *et al*. 2010). However, in many rangelands outside of protected areas, the addition of livestock is commonly associated with wildlife decline. Particularly in Africa, where > 60% of the continent is savanna, where humans and livestock have coexisted with wildlife for thousands of years and where livestock densities (especially small stock) continue to increase, it is especially important to consider wildlife decline in conjunction with changes in livestock abundance.

Our results demonstrate that the changes in plant communities in response to declines of native herbivores in experimental sites do not closely approximate the changes that occur in plant communities in more typical landscapes in this region in which livestock have replaced wildlife. For all structural metrics examined, experimental vs. landscape status was one of the most important factors identified in predicting variation in plant structure. It also had the strongest interactions with wildlife declines in predicting structural responses to herbivore declines, causing reversals in the direction of effects of plant response to wildlife decline for all plant metrics examined (Fig. 2). In experimental sites, we noted strong increases in aerial cover, height and total cover of vegetation and subtle increases in species richness with the loss of wildlife; these responses were inverted in landscape sites, probably due to livestock herbivory.

Effects of wildlife loss on community composition and type of growth form present were likewise highly divergent between experimental and landscape sites. While there were no significant changes in community composition with wildlife declines in the experimental sites, there were strong changes in composition in the surrounding landscape (decrease in abundance of forbs and plants with woody growth forms in low wildlife environments) where wildlife decline was often associated with livestock increases. Overall, livestock abundance was much more important than wildlife abundance in determining plant community composition. Livestock superseded even environmental variables in determining growth form composition, and landscape sites in which livestock dominated were distinct both in species composition and growth form composition. Livestock-dominated experimental sites did not closely approximate livestock-dominated landscape sites, likely due in part to differences in stocking density, which is often extremely high (i.e. overstocking) in landscape sites. Indeed, total herbivore abundance in experimental sites never approached the high value levels seen in landscape sites, in large part because of very high stocking densities of livestock at some landscape sites. However, even within the range of total herbivore abundance seen in both experimental and landscape sites, plant community characteristics did not show similar responses to changes in herbivore abundance across these treatments (e.g. Fig. 2).

The magnitude and shape of the relationship between abiotic drivers and herbivore effects also varied between experimental and landscape sites. In the experimental sites, increased rainfall and reduced sand:silt ratios led to lower effects of herbivore loss on plant communities. However, in the surrounding landscape sites, the sand:silt content had much reduced or no effect on structure parameters. Wildlife declines in landscape sites were unimodally related to plant structure and species richness with rainfall, with strong effects of wildlife decline in low and high rainfall environments and much reduced effects at intermediate rainfall levels (Fig. 3 and Fig. S2). This unimodal effect is consistent with that seen across a larger rainfall gradient in the Serengeti (Anderson, Ritchie & McNaughton 2007). In that system, part of the reason for the unimodal effect was likely to be the reciprocal interactions between wildlife and soil characteristics (with wildlife declines driving changes in soil nutrients, which then drove changes in plant communities).

Notably, there were strong and systematic differences among landscape and experimental sites despite considerable variation within each site type. For example, the two experiments (KLEE and UHURU) examined differed in many ways, including the duration of the experiment (4 vs. 17 years at time of sampling), the underlying soil properties, the particular size class of herbivores excluded and the identity of dominant herbivores excluded. Landscape sites also varied in the time period over which domestic stock had been introduced, the composition of the domestic stock introduced and the degree of other human disturbances in the landscape.

### Herbivore identity and compensation

The variation in plant community composition, species richness, and structure between experimental and landscape sites is probably due in large part to replacement of wildlife with livestock in landscape but not (most) experimental sites. Some of this variation is probably caused by variation in absolute herbivore stocking density between experimental and landscape sites. Isolated removal of wild herbivores would naturally cause different effects on plant communities than would their replacement with livestock. Further, large herbivores often show strong changes in the selectivity of their diet at high stocking densities. Combined with simple increases in leaf area removed at higher densities, these changes in feeding patterns can cause highly divergent effects on plant diversity and structure depending on their stocking rates (Augustine & McNaughton 1998). High stocking densities and overstocking is an issue in many ranches in East Africa (Lamprey & Reid 2004), and many pastoral landscapes in the study region have relatively high livestock densities (> 25 cattle equivalents km^−2^) that greatly exceed stocking densities in wildlife-dominated natural landscapes (Georgiadis *et al*. 2007; Kinnaird & O'Brien 2012). However, the difference in total stocking levels does not appear to be the sole driver of patterns observed here.

The effects of experimental status on plant community response to herbivore declines were similarly strong when we used relative abundance of all herbivores as opposed to relative abundance of wild herbivores only (Table S3). Experimental and landscape sites with similar relative stocking densities of herbivores thus often had very different plant communities after controlling for abiotic factors (i.e. Fig. 2). These results may point to strong differences in effects of wildlife vs. livestock on plant communities, perhaps due to imperfect diet compensation or other behavioural differences among the groups (i.e. physical damage by elephants). The feeding niches of native large mammals in Laikipia include a mixture of grazing, browsing and mixed feeding. Likewise, the domestic livestock that replace them also have a mixture of feeding strategies – cattle and sheep are primarily grazers, and goats and camels are browsers. It is thus unlikely that differences in feeding niches between native ungulates and the domestic wildlife that replace them was the primary driver of our results. However, there are likely to be changes in relative abundance of grazers vs. browsers as livestock are introduced, as well as subtle changes in diet selectivity and associated changes in the average size class of herbivores. In particular, the loss of the largest of wild herbivores, and replacement by smaller livestock species, will result in a reduction in mean body size of consumers, generally resulting in greater diet selectivity but lower biomass removal (Damuth 1981; Bakker *et al*. 2006). However, at intermediate stocking densities, the differences between landscape and experimental sites are minimized, suggesting that at these intermediate levels domestic stock may compensate fairly well for wild herbivores.

These results may also point to other differences between experimental and landscape sites not accounted for by differences in stocking density, or in variation between impacts of wildlife and livestock including experimental artefacts, and other direct human alterations to plant communities in low wildlife or high livestock landscapes. For example, charcoaling activities by humans that often occur in livestock-dominated sites (Okello, O'Connor & Young 2001) may explain declines in woody vegetation in landscape sites. In landscape sites, it is also difficult to identify the causal direction of the relationship between wildlife, vegetation and livestock. While in experimental sites the number of herbivores (either livestock or wild herbivores) clearly drives the vegetation parameters, in the landscape sites, differences in vegetation may either be driven by livestock abundance, and secondarily influence wildlife abundance (i.e. livestock supplant wildlife) or may be driven by the combination of wildlife decline and subsequent livestock compensation. In most managed landscapes, it seems likely that both of these phenomena are at play.

## Conclusions

In African savannas, the loss of large wild herbivores has been demonstrated to lead to myriad changes in vegetation diversity (Augustine & McNaughton 1998; Anderson, Ritchie & McNaughton 2007); faunal communities, including rodents (Keesing 1998), reptiles (McCauley *et al*. 2006) and insects (Pringle *et al*. 2007); plant–animal interactions (Palmer *et al*. 2008); nutrient dynamics (Augustine 2003); and ecosystem stability (Goheen & Palmer 2010). The consequences of the loss of the large wild herbivore guild are thus complex and cannot be effectively considered in isolation from the responses of other taxa or environmental context (McNaughton 1983; Davidson *et al*. 2010). This study highlights the strong and interacting effects of both environmental gradients and degree of replacement or compensation by livestock in predicting the effects of such declines in wild native herbivores, at least on plant communities. Given ongoing regional and global transformations to fundamental resource gradients (i.e. nutrients and rainfall), wildlife declines (Ottichilo *et al*. 2000) and continued proliferation and intensification of livestock and ranching activities (Prins 2000; Lamprey & Reid 2004), we will need to explicitly incorporate both these factors in our analyses in order to accurately understand the most probable outcomes of wildlife decline in African savanna landscapes and beyond.

Experimental exclosures and herbivore manipulations will continue to be a critical part of understanding and isolating the ecological role of large wild mammals. However, to truly understand the likely ecological outcome of declines of native herbivores in African savanna landscapes and beyond, we will need to more regularly include in our toolset experimental manipulations that include compensation from livestock at varying intensities. While such studies exist, including (to some extent) the KLEE experiment used here, they are uncommon and often still do not fully account for changes in plant communities observed in typically low wildlife landscapes. We will also need to continue to compare results from experimental results with larger-scale comparative studies in surrounding landscapes in order to better understand the applications of experimental manipulations to management and conservation choices.

Results from this study and others suggest that the trade-offs between livestock and wild herbivores may be complex. Competition or compensation between livestock and wildlife is likely to vary based on environmental gradients, stocking densities, species assemblages and management strategies. Environmental gradients, in particular, which are known to play strong roles in determining plant responses to herbivory, appear to have more muted effects when livestock are allowed to compensate, compared to when only wildlife herbivore abundance is considered. In order to better identify, predict and ameliorate the changes in communities that are likely to occur under realistic scenarios of wildlife declines, we will need more well-replicated studies that explicitly compare effects of wildlife to those of livestock on the ecological communities in which these species are embedded.

## Supporting Information

**Appendix S1.** Validation of dung surveys as an index of relative abundance of herbivores.

**Appendix S2.** Effects of wildlife on soil properties.

**Appendix S3.** Analysis of spatial autocorrelation in plant community composition data.

**Figure S1.** Relationship between estimates of wildlife and domestic stock from dung surveys to those from camera-trap surveys.

**Figure S2.** Effect sizes (log_e_ response ratios) of wildlife removal on structural characteristics of the plant community across abiotic gradients.

**Figure S3.** Relationship between soil texture, livestock abundance and plant species richness.

**Figure S4.** Mantel correlogram plots showing spatial autocorrelation in data before and after transformations.

**Figure S5.** Results from discrimination analysis of plant community composition after controlling for spatial autocorrelation.

**Table S1.** Summary of analytical approaches used.

**Table S2.** Model average parameters for models of plant community characteristics based on abundance of all herbivores and selected environmental factors.

**Table S3.** Model average parameters for models examining the effect size (log_e_ response ratios) of herbivore effects on plant communities by selected environmental gradients.
