# Supplementary material for: Effects of mammalian herbivore declines on plant communities: observations and experiments in an African savanna
Source: J Ecol. 2013 Jun 6;101(4):1030–41. doi: 10.1111/1365-2745.12096 (PMC3758959; doi:10.1111/1365-2745.12096)
Supplement: Supplementary file 1 [file jec0101-1030-SD1.doc]

**SUPPORTING INFORMATION**

Additional supporting information may be found in the online version of this article:

**Appendix S1** *Validation of dung surveys as an index of relative abundance of herbivores*

**Appendix S2** *Effects of wildlife on soil properties*

**Appendix S3** *Analysis of spatial autocorrelation in plant community composition data*

**Figure S1** *Relationship between estimates of wildlife and domestic stock from dung surveys to those from camera trap surveys.*

**Figure S2** *Effect sizes (log_e_ response ratios) of wildlife removal on structural characteristics of the plant community across abiotic gradients*

**Figure S3** *Relationship between soil texture, livestock abundance and plant species richness*

**Figure S4** *Mantel correlogram plots showing spatial autocorrelation in data before and after transformations.*

**Figure S5** *Results from discrimination analysis of plant community composition after controlling for spatial autocorrelation*

**Table S1** *Summary of analytical approaches used*

**Table S2** *Model average parameters for models of plant community characteristics based on abundance of all herbivores and selected environmental factors*

**Table S3** *Model average parameters for models examining the effect size (log_e_ response ratios) of herbivore effects on plant communities by selected environmental gradients*

**APPENDIX S1:** *Validation of dung surveys as an index of relative abundance of herbivores*

There is a wide variety of wild herbivores in this region, including elephants (*Loxodonta africana*), giraffes (*Giraffa camelopardalis*), zebras (*Equus burchelli* and *Equus grevyi*), buffalo (*Syncerus caffer),* impala (*Aepyceros melampus*), Grant’s gazelles (*Nanger [Gazella] granti*), eland (*Taurotragus oryx*), and hartebeest (*Alcelaphus buselaphus*), among others. Common livestock species include cattle (*Bos primigenius*), goats (*Capra hircus*), sheep (*Ovis aries*), and camels (*Camelus dromedarius*). Calibrating any index of animal abundance to true densities is challenging, particularly when, as in this system animal communities are diverse. Obtaining true density estimates is thus not a goal of this study. Instead we are intending to only estimate relative abundance of animals across sites. The primary metric we use is abundance of dung of each species. However, dung surveys have several shortcomings. Due to highly variable digestive systems among ungulate taxa, and very different rates of decay of dung material, dung abundance can be highly variable among species and across sites (Kuehl *et al.* 2007). To validate our dung estimates of animal abundance from dung surveys we also used complementary camera trapping data at a subset of 61 of our sites. While camera trapping is often used to detect presence/ absence of rare species and to estimate abundance using individual recognition, camera trap photographic rates (photographs per unity sampling time) can be used to estimate abundance without any need for individual recognition of animals (O'Brien *et al.* 2003; Rovero & Marshall 2009).

Our camera trapping efforts used a combination of Reconyx (RM45) and Scout Guard digital cameras (Model SG750; HCO, Norcross, GA). Two cameras were placed at or within 100 m of each site. Cameras were positioned at least 50 m apart and specific locations were selected using presence of animal trails and dung piles to maximize likely detection. Cameras were set to take pictures 24 hours a day, with a 30 second delay between each three images. Date and time were recorded for each image taken. Cameras were left in the field for up to 21 days per site. All mammals in each image were identified and counted, but for consecutive photographs of the same species in the same species we simply counted the maximum number of animals of that species within a 0.5 hour interval (Bowkett *et al.* 2007). Camera trapping rate was defined as the number of animals observed per hour. All animals were considered wild animals except for camels, goats, sheep, and cattle, which were classified as domestic livestock, and humans, which were not considered in this analysis.

While it was possible to obtain over 1000 camera trapping hours per site with these methods, due to various logistic difficulties (i.e. card was filled, camera malfunctions or was stolen, or batteries died), only 30 sites surveyed actually had more than 1000 camera trap hours. We excluded 11 sites which had less than 200 camera trap hours total. We compared camera trap from the remaining 50 sites results using linear regression. We found a strong correlation between estimates of livestock (R^2^ =0.50, P <0.0001) and wildlife (R^2^ = 0.30, P < 0.0001) relative abundance obtained via this camera trap analysis and the estimates obtained via dung surveys (Fig S1). Overall, dung estimates appear to provide a slightly lower estimate of actual prevalence of animals (as assessed by camera traps) for domestic stock than for wildlife.

**S1 References**

Bowkett, A.E., Lunt, N., Rovero, F. & Plowman, A.B. (2006) How do you monitor rare and elusive mammals? Counting duikers in Kenya, Tanzania and Zimbabwe. In: Zgrabczynska, E., Scwiertnia, P. and Ziomek, J. (eds.), Animals, Zoos, and Conservation, pp. 21-28.

Kuehl, H.S., Todd, A., Boesch, C. & Walsh, P.D. (2007) Manipulating decay time for efficient large-mammal density estimation: gorillas and dung height. *Ecological Applications,* **17**, 2403-2414.

O' Brien, T.G., Kinnaird, M.F. & Wibisono, H.T. (2003) Crouching tigers, hidden prey: Sumatran tiger and prey populations in a tropical forest landscape. *Animal Conservation,* **6,** 131-139.

Rovero, F. & Marshall, A.R. (2009) Camera trapping photographic rate as an index of density in forest ungulates. *Journal of Applied Ecology,* **46**, 1011-1017.

**APPENDIX S2:** *Effects of wildlife on soil properties:*

Multiple studies have documented that high densities of large wildlife and livestock can drive changes in environmental gradients, particularly soil nutrients, thereby influencing plant community composition, diversity, and structure (e.g. Augustine & McNaughton 2006). To account for such potential effects of wildlife on soil properties, we also examined the differences in soil properties among paired sites. At each experimental and landscape site, an integrated soil sample of 20-cm depth was taken at 3-5 locations per site. Samples were subsequently dried (65 °C), after which all samples from each site were homogenized and sieved through 2-mm mesh. Soils were sent for analysis to Brookside Laboratories (New Knoxville, OH). Structural and chemical characteristics examined include: pH, organic matter (based on loss on ignition); percent sand, silt, and clay; extractable mineral N (nitrate and ammonium, using KCl extractions); extractable phosphate (using Bray 1 extractions), and extractable Ca, Mg, and K. We used the GLMs of log response ratio on paired sites only (as described in main text) to examine possible effects of wildlife on pH, percent organic matter, plant available P, NO_3_^-^ and NH_4_^+^ and extractable Ca, Mg, and K.

We found no significant relationship between high wildlife and low wildlife sites and any soil property examined in either the experimental or landscape sites (paired t-tests between sites, P > 0.17 for all soil properties). Further, soil, rainfall, and experimental status (and their interactions) had no significant explanatory power in driving variation in soil property responses to herbivore declines across sites (P > 0.24 for all soil properties). It seems likely that underlying edaphic variation in nutrient content overpowered the potential effects of variation in wildlife abundance on these properties.

**APPENDIX S3:** *Analysis of spatial autocorrelation in plant community composition data*

In order to account for the potential effects of spatial autocorrelation on the observed multivariate floral communities, we assessed the magnitude of the spatial effect by calculating and testing a Mantel correlogram ([Mantel 1967](https://mail.google.com/mail/u/0/html/compose/static_files/blank_quirks.html#_ENREF_4); [Oden and Sokal 1986](https://mail.google.com/mail/u/0/html/compose/static_files/blank_quirks.html#_ENREF_5); [Sokal 1986](https://mail.google.com/mail/u/0/html/compose/static_files/blank_quirks.html#_ENREF_6)). The Mantel statistic is computed at sequential distance lags between a community dissimilarity matrix and a design matrix of 1s and 0s (see [Legendre and Legendre 1998](https://mail.google.com/mail/u/0/html/compose/static_files/blank_quirks.html#_ENREF_3)). The multivariate Mantel correlogram test has been shown to be a very powerful technique to detect multivariate spatial structure ([Borcard and Legendre 2012](https://mail.google.com/mail/u/0/html/compose/static_files/blank_quirks.html#_ENREF_1)).

First, we computed a dissimilarity matrix between floral communities using the Canberra metric (see methods section). Following computation of the Mantel correlogram, and significant testing through permutation, we see that significant positive spatial autocorrelation is present at lags up to 14.7 km (Fig S4a). We then normalized the data using the Hellinger transformation ([Legendre and Gallagher 2001](https://mail.google.com/mail/u/0/html/compose/static_files/blank_quirks.html#_ENREF_2)); here distances between communities can be calculated in Euclidean space. We then computed the Mantel correlogram and tested for significant spatial autocorrelation. Statistically significant spatial autocorrelation results were very similar to original data matrix (Fig S4b).

To account for the observed spatial autocorrelation, we regressed the (hellinger transformed) community data at each site against their respective latitude and longitude-coordinates. Residuals of this regression were then plotted and tested again using a Mantel correlogram to assess how well these residuals were decoupled of their spatial component.  The Mantel correlogram plot illustrates the coordinate regressed residuals now only showing spatial autocorrelation at very small scales: at a distance of 0.015 decimal degrees and shorter (distances ≤ 1.11 km; Fig S4c). Only paired sites were at this close of proximity (which was intended to control for effects of variation in soil and rainfall characteristics).  We then used these residuals, which had very little spatial structure to recompute ordinations and vector fitting analysis described in main text for plant community composition.

As would be anticipated given that that both rainfall and soil gradients occur over spatial gradients (rainfall in particular has a strong north-south gradient in this system), we find a strong degree of spatial autocorrelation in our dataset. However, when we remove the spatial autocorrelation, we find that the conclusions of the analyses presented in the main text remain largely unchanged (Fig S5). Strong differences in composition can be seen between high wildlife and low wildlife levels in landscape but not experimental sites; this relationship seems to be due largely to the increase in livestock in low wildlife sites in landscape sites.  Wildlife itself is not a significant explanatory variable, but livestock abundance is strongly significant. Important environmental drivers of plant community composition include the underlying soil parameters (% sand, silt, and clay).  The only qualitative difference in these results from those presented in the main text (without controlling for spatial autocorrelation) is that rainfall, which is highly spatially autocorrelated, changes from becoming highly significantly predictive of plant community composition (P<0.01) in main analysis to marginally predictive of plant community composition (P=0.09) in analyses that control for spatial autocorrelation.

**S3 References**

Borcard, D., & Legendre, P. (2012) Is the Mantel correlogram powerful enough to be useful in ecological analysis? A simulation study. *Ecology,* **93,** 1473-1481.

Legendre, P., & Gallagher, E.D. (2001) Ecologically meaningful transformations for ordination of species data. *Oecologia*, **129,** 271-281.

Legendre, P. & Legendre, L. (1998) Numerical Ecology, 2nd ed. Elsevier, New York.

Mantel, N. (1967) The detection of disease clustering and a generalized regression approach. *Cancer Research,* **27,** 209-220.

Oden, N.L., & Sokal, R.R. (1986). Directional autocorrelation - an extension of spatial correlograms to 2 dimensions. *Systematic Zoology,* **35,** 608-617.

Page, J., Schektman, Y., & Tomassone, R. (1986) Data Analysis and Informatics IV. Proceedings of the Fourth International Symposium of data analysis and informatics, Versailles, France. pp 29-43.

**Table S1:**  Overview of analytical approaches and sample sizes used

| **Analysis** | **Response variables** | **Model details** | **n** |
| --- | --- | --- | --- |
| GLM unpaired | Height | Gaussian errors and identity log link | 74 (27 experimental, 47 landscape) |
|  | Aerial cover | Gaussian errors and identity log link | 74 (27 experimental, 47 landscape) |
|  | Total cover | Gaussian errors and identity log link | 74 (27 experimental, 47 landscape) |
|  | Species richness | Poisson errors and log link functions | 74 (27 experimental, 47 landscape) |
| GLM paired | Height log response ratio | Gaussian errors and identity log link | 24 pairs (12 experimental, 12 landscape) |
|  | Aerial cover log response ratio | Gaussian errors and identity log link | 24 pairs (12 experimental, 12 landscape) |
|  | Total cover log response ratio | Gaussian errors and identity log link | 24 pairs (12 experimental, 12 landscape) |
|  | Species richness log response ratio | Poisson errors and log link functions | 24 pairs (12 experimental, 12 landscape) |
| NMDS with nonparametric multivariate ANOVA | community composition - species level | | 74 (27 experimental, 47 landscape) |
|  | community composition - life form level | | 74 (27 experimental, 47 landscape) |

**Table S2:** Model average parameter estimates including standard errors (SE), relative variable importance, and estimated *P* values. Analyses are based on entire data set of 74 sites, with plant data pooled at the site level. *P* values are determined using backwards stepwise regression from the full model. In this analysis total abundance of herbivores is considered jointly rather than separated into domestic and wildlife (as in Table 1).

| **A. Vegetation Height** | Coeff ± SE | relative importance | Pr(>|z|) |
| --- | --- | --- | --- |
| (1) Main effects |  |  |  |
| Herbivore abundance | -6.68 ± 2.19 | 1.00 | **<0.01** |
| Experimental status | -15.83 ± 2.83 | 1.00 | **<0.001** |
| Annual rainfall | 0.041 ± 0.001 | 1.00 | **<0.001** |
| Soil (sand:silt ratio) | -- | -- | -- |
| Recent rainfall | 2.98 × 10^-3^ ± 1.65 × 10^-2^ | 0.19 | n.s |
| (2) Selected interactions |  |  |  |
| Experiment × herbivores | 7.09 ± 1.89 | 1.00 | **<0.001** |
| Rainfall × herbivores | 6.54 × 10^-5^ ± 5.41 × 10^-3^ | 0.18 | n.s |
| Soil × herbivores | -- | -- | -- |
| **B. Aerial Cover** |  |  |  |
| (1) Main effects |  |  |  |
| Herbivore abundance | -0.13 ± 0.06 | 0.90 | 0.04 |
| Experimental status | -0.26 ± 0.01 | 1.00 | **<0.01** |
| Annual rainfall | 1.35 × 10^-3^ ± 2.43 × 10^-4^ | 1.00 | **<0.001** |
| Soil (sand:silt ratio) | -- | -- | -- |
| Recent rainfall | 3.393× 10^-6^ ± 3.26 × 10^-4^ | 0.17 | n.s |
| (2) Selected interactions |  |  |  |
| Experiment × herbivores | 0.15 ± 0.05 | 1 | **<0.01** |
| Rainfall × herbivores | 3.37 × 10^-5^ ± 1.51 × 10^-4^ | 0.17 | n.s |
| Soil × herbivores | -- | -- | -- |
| **C. Total Cover** | |  |  |
| (1) Main effects |  |  |  |
| Herbivore abundance | -2.16 ± 0..82 | 1.00 | **<0.001** |
| Experimental status | -5.94 ± 0.93 | 1.00 | **<0.001** |
| Annual rainfall | 0.02 ± 0.003 | 1.00 | **<0.001** |
| Soil (sand:silt ratio) | -- | -- | -- |
| Recent rainfall | -6.36 × 10^-4^ ± 3.83 × 10^-3^ | 0.18 | n.s |
| (2) Selected interactions |  |  |  |
| Experiment × herbivores | 2.41 ± 0.62 | 1.00 | **<0.001** |
| Rainfall × herbivores | -1.34 × 10^-3^ ± 1.77 × 10^-3^ | 0.24 | n.s |
| Soil × herbivores | -- | -- | -- |

**Table S3:** Model average parameters for models examining the effect size (log_e_ response ratios) of herbivore effects on plant communities by various environmental gradients. Estimates include standard errors (SE), and relative variable importance. Analyses are based only on the subset of sites that were spatially and temporally paired across a strong gradient of wildlife abundance. Data is pooled at the pair level (n=24 pairs).

|  | **Aerial Cover** | |  | **Average Height** | |
| --- | --- | --- | --- | --- | --- |
|  | Coeff ± SE | relative importance |  | Coeff ± SE | relative importance |
| Experimental status | -2.922 ± 3.722 | 1.00 |  | 40.21 ± 84.42 | 1.00 |
| Annual rainfall | -1.105 ± 0.983 | 0.36 |  | -1.474 ± 1.628 | 0.63 |
| (Annual rainfall)^2^ | 3.3 × 10^-4^ ± 3.1 × 10^-4^ | 0.38 |  | 4.64 × 10^-4^ ± 5.20 × 10^-4^ | 0.62 |
| Soil (sand:silt ratio) | 0.080 ± 0.081 | 0.18 |  | 0.127 ± 0.162 | 0.10 |
| Experimental × rainfall | 7.62 × 10^-6^ ± 4.20 × 10^-6^ | 0.18 |  | 0.095 ± 0.286 | 0.30 |
| Experimental × (rainfall)^2^ | 6.643 × 10^-6^ ± 3.516 × 10^-6^ | 0.18 |  | 1.01 × 10^-4^ ± 8.96 × 10^-5^ | 0.31 |
| Experimental × soil | -0.231 ± 0.112 | 0.13 |  | -0.165 ± 0.186 | 0.01 |
|  |  |  |  |  |  |
|  |  |  |  |  |  |
|  | **Total Cover** | |  | **Species Richness** |  |
|  | Coeff ± SE | relative importance |  | Coeff ± SE | relative importance |
| Experimental status | 84.32 ± 402.70 | 1.00 |  | 69.1 ± 324.1 | 1.00 |
| Annual rainfall | -1.824 ± 1.765 | 0.60 |  | -0.836 ± 0.884 | 0.53 |
| (Annual rainfall)^2^ | 5.64 × 10^-4^ ± 5.68 × 10^-4^ | 0.60 |  | 2.70 × 10^-4^ ± 2.84 × 10^-4^ | 0.52 |
| Soil (sand:silt ratio) | 0.203 ± 0.134 | 0.09 |  | 3.54 × 10^-3^ ± 1.12 × 10^-1^ | 0.22 |
| Experimental × rainfall | 0.639 ± 2.408 | 0.33 |  | 1.131 ± 2.355 | 0.20 |
| Experimental × (rainfall)^2^ | 7.25 × 10^-5^ ± 7.17 × 10^-4^ | 0.35 |  | 2.71 × 10^-4^ ± 6.83 × 10^-4^ | 0.22 |
| Experimental × soil | -0.312 ± 0.201 | 0.50 |  | -0.197 ± 0.121 | 0.11 |

**Fig S1:**


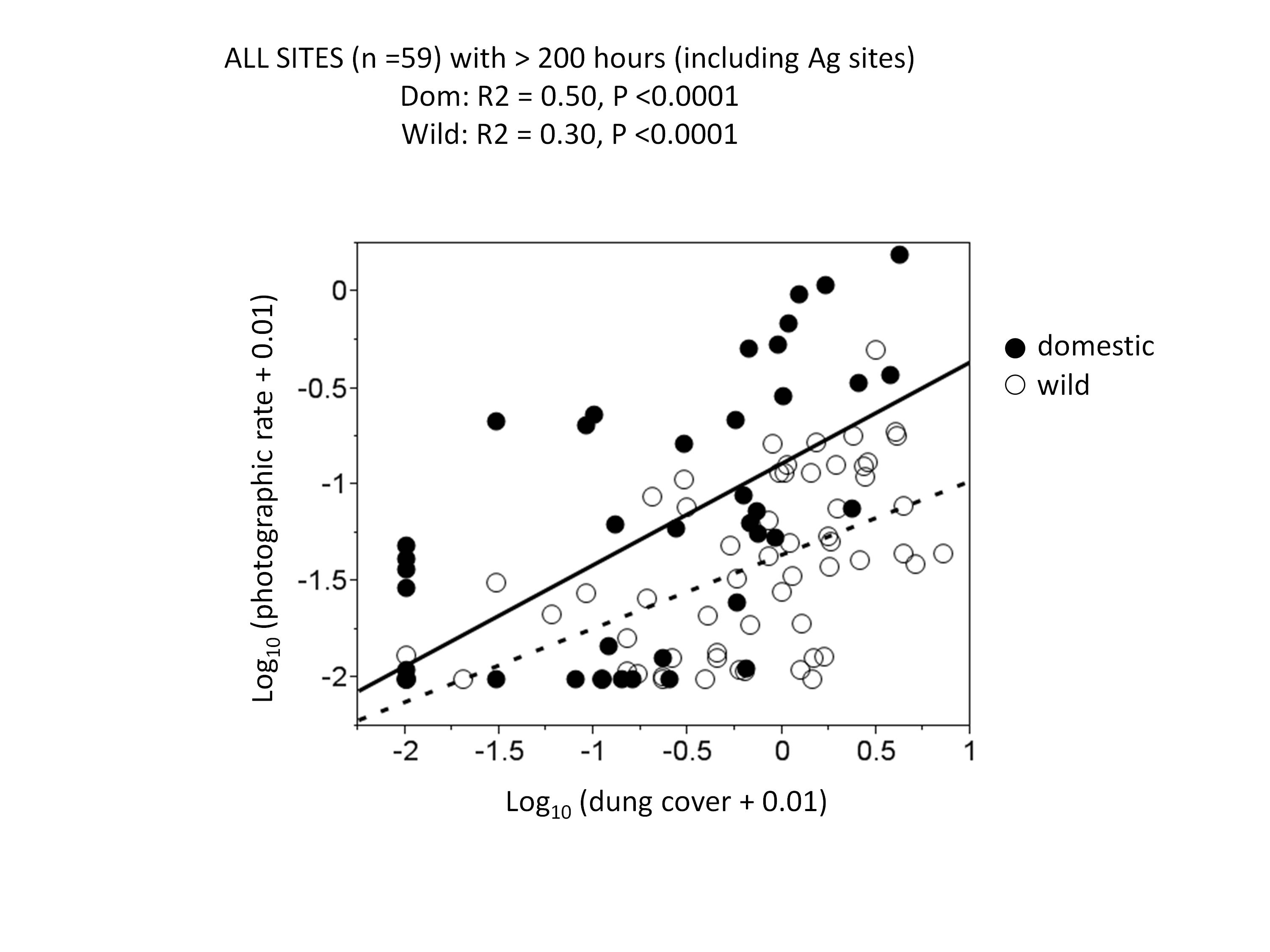


The percent cover of wildlife dung (unfilled circles) and domestic stock (filled circles) dung are both strongly correlated to abundance as estimated by camera traps, although dung appears to provide a slightly lower estimate of animal abundance for domestic as for wildlife species

**Fig S2**:


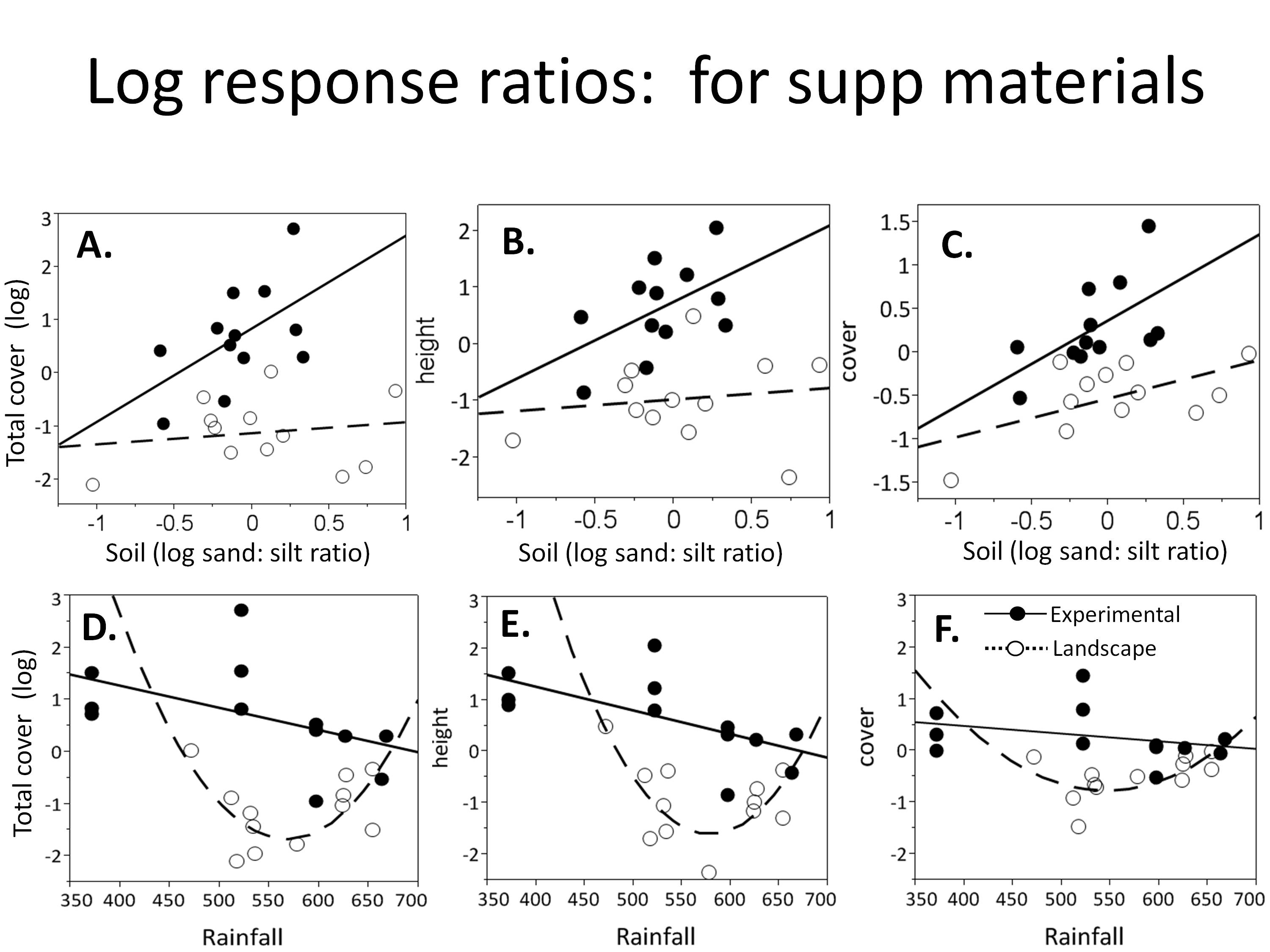


Effect sizes (log_e_ response ratios) of wildlife removal on three structural characteristics of the plant community (total cover, height, and % aerial cover) across a gradient of soil sand:silt ratios (where lower sand:silt ratios are characteristic of high productivity black cotton soils) (A-C) and a gradient of rainfall (mean annual rainfall) (D-F). For all three structural metrics the effects of wildlife loss causes a stronger response in less productive soil environments (high sand:silt ratios; A-C); but this effect is muted in the surrounding landscapes (dashed line, open circles) as compared to experimental sites (solid black line, filled circles). Increasing rainfall was associated with slightly lower responses to wildlife loss in experimental sites and a mild unimodal response in landscape sites (lower responses at intermediate levels of rainfall). Only the subset of sites that were spatially and temporally paired were used for these analyses (24 pairs). All lines represent the best fit from model comparison analyses.

**Fig S3:**


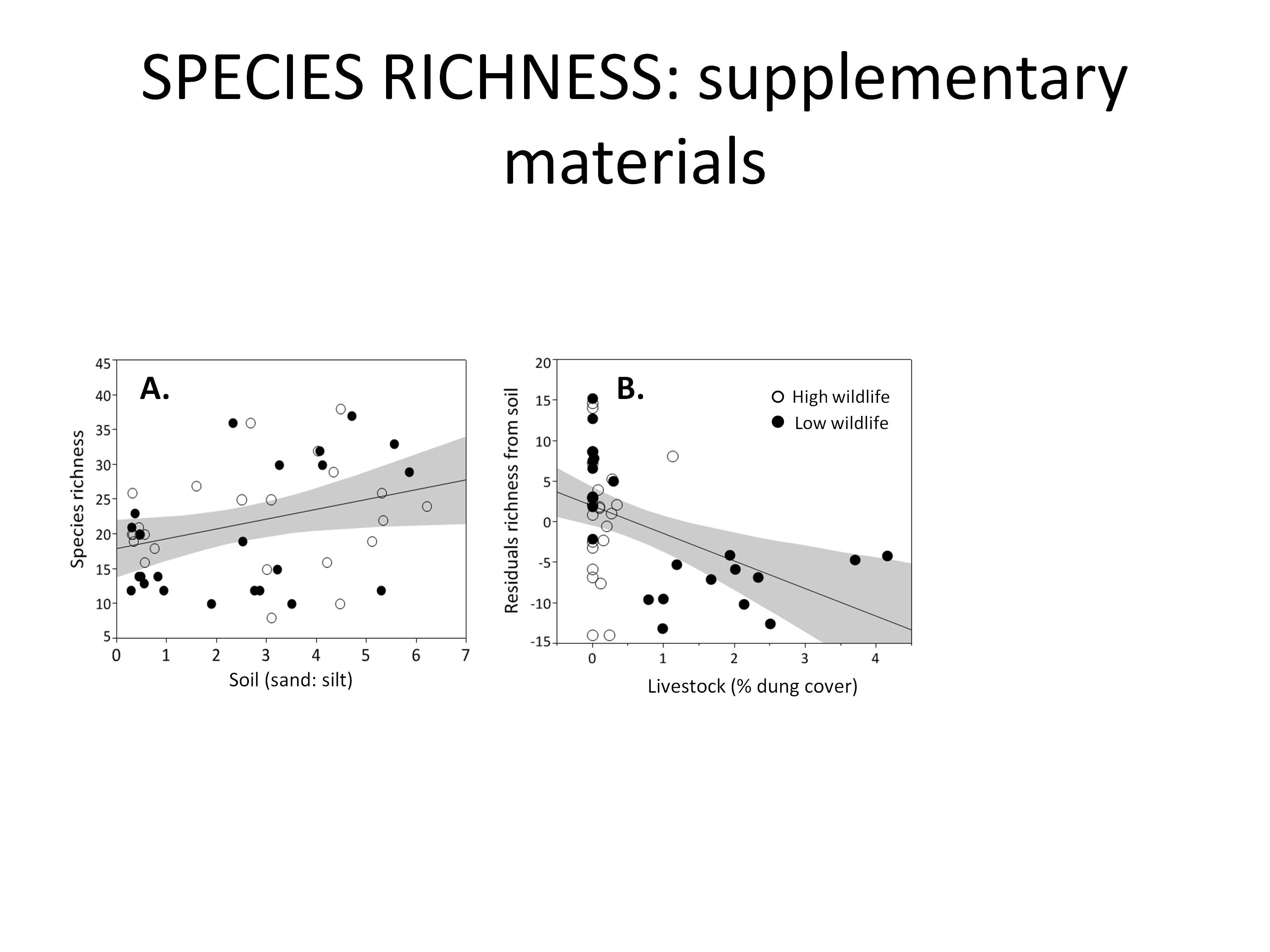
Models of plant species richness show a strong effect of soil parent material, with more species rich plant communities found on lower productivity red soil sites (A). There is no clear effect of abundance of wildlife on plant species richness in these analyses, but livestock abundance does explain much (~20%) of the residual variation. The grey shaded area represents the 95% confidence interval for response across all sites.

**Fig S4:

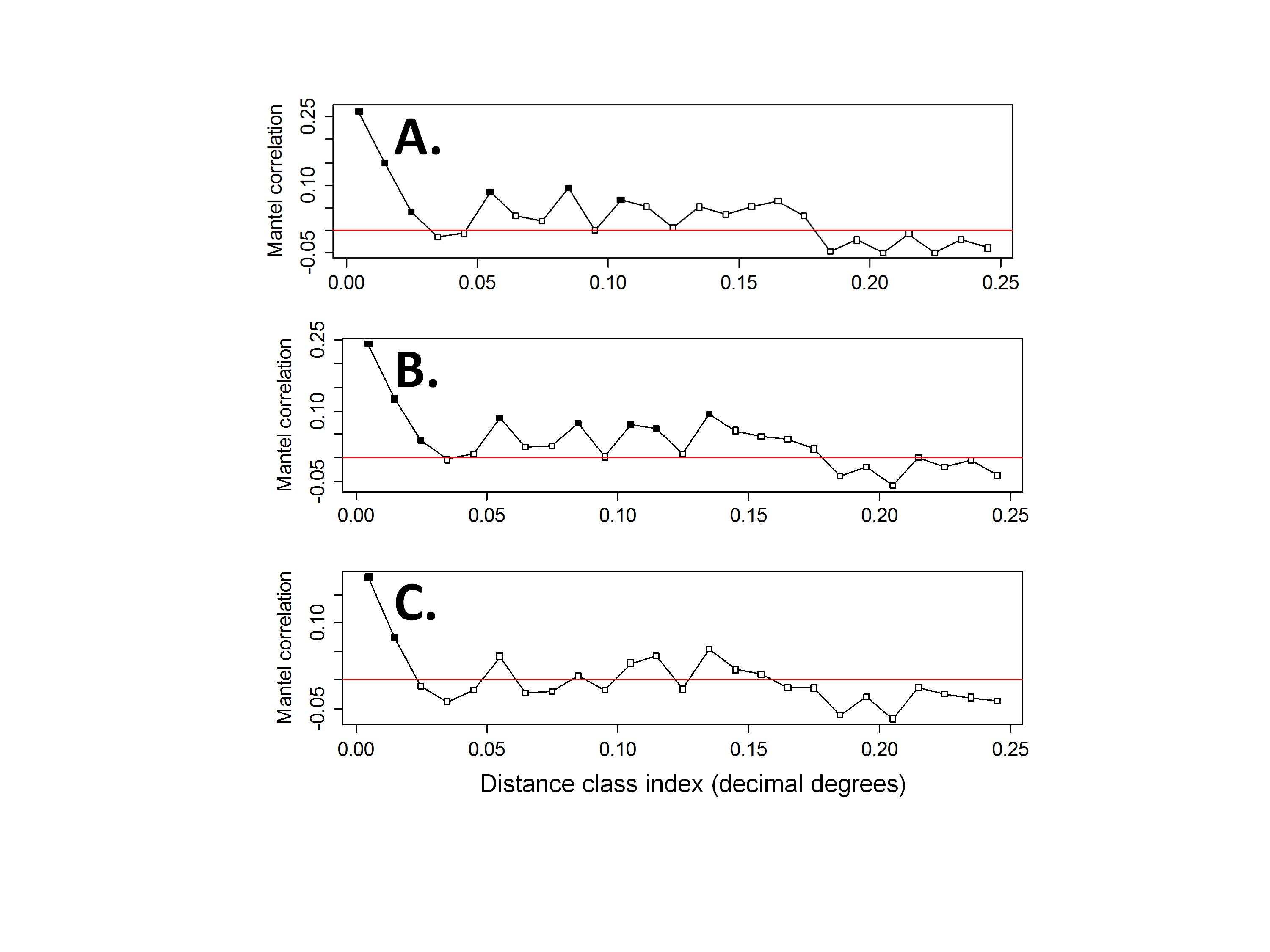
**

Mantel correlogram plot shows that there was initially strong spatial autocorrelation in data, at distances up to 0.11 decimal degrees (0.11 km; Panel A). Results after Hellinger transformation are largely similar (B). However, after regressing the community data at each site against their respective latitude and longitude (C), the Mantel correlogram shows that the residuals have spatial autocorrelation only at very small spatial scales of 0.015 decimal degrees and shorter (1.11 km). In this figure significant spatial autocorrelations are shown in filled squares, hollow squares represent nonsignificant spatial autocorrelation.

**Fig S5:***
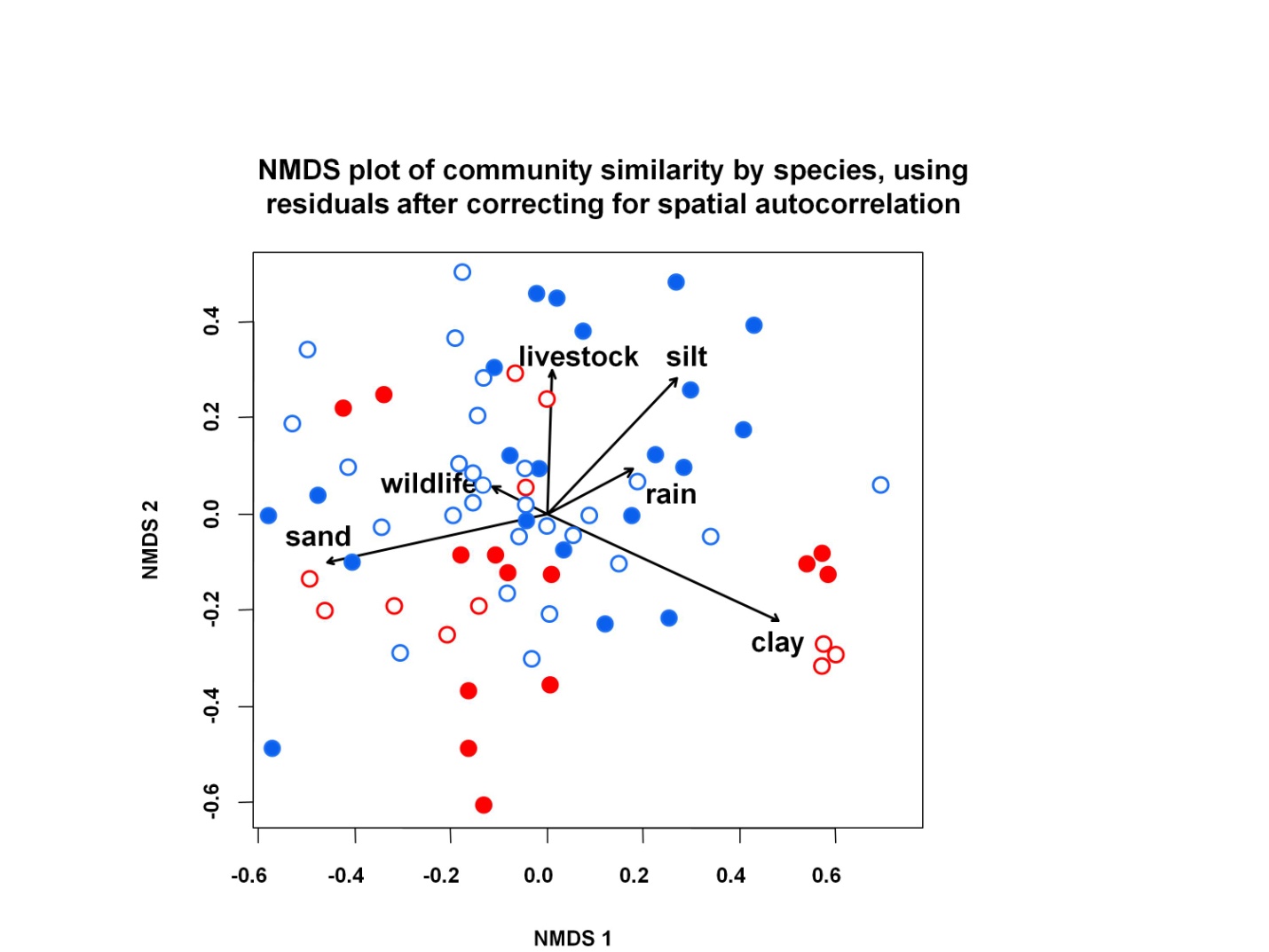
*

As in analyses presented in main text without controlling for spatial autocorrelation, there are strong and consistent differences in composition exist between high wildlife (unfilled circles) and low wildlife sites (filled circles) in landscape sites (in blue) but not in experimental sites (in red). Important environmental drivers of plant community composition (shown with black arrows) are underlying soil parameters (% sand, silt, and clay), and domestic livestock. Wildlife is not a strong predictor of plant communities. The only qualitative difference in these results from those in the main text is that rainfall changes from becoming highly significantly predictive of plant community composition (P<0.01) to marginally predictive of plant community composition (P=0.09).
